# Metagenomic Approaches Reveal Strain Profiling and Genotyping of Klebsiella pneumoniae from Hospitalized Patients in China

**DOI:** 10.1128/spectrum.02190-21

**Published:** 2022-03-23

**Authors:** Jiao Liu, Zhuofei Xu, Haibo Li, Fuhui Chen, Kaiyu Han, Xiaoman Hu, Yuan Fang, Dechang Chen

**Affiliations:** a Department of Critical Care Medicine, Ruijin Hospital, Shanghai Jiao Tong University School of Medicine, Shanghai, China; b Genoxor Medical Science and Technology Inc., Zhejiang, China; c The Central Laboratory of Birth Defects Prevention and Control, Ningbo Women and Children’s Hospital, Ningbo, China; d Department of Respiration, the Second Affiliated Hospital of Harbin Medical University, Harbin, China; Huazhong University of Science and Technology

**Keywords:** *K. pneumoniae*, metagenome-reconstructed strains, strain profiling, phylogeny, MLST, capsule typing, antimicrobial resistance genes, virulence genes

## Abstract

Klebsiella pneumoniae is a leading cause of highly drug-resistant infections in hospitals worldwide. Strain-level bacterial identification on the genetic determinants of multidrug resistance and high pathogenicity is critical for the surveillance and treatment of this clinically relevant pathogen. In this study, metagenomic next-generation sequencing was performed for specimens collected from August 2020 to May 2021 in Ruijin Hospital, Ningbo Women and Children’s Hospital, and the Second Affiliated Hospital of Harbin Medical University. Genome biology of K. pneumoniae prevalent in China was characterized based on metagenomic data. Thirty K. pneumoniae strains derived from 14 sequence types were identified by multilocus sequence typing. The hypervirulent ST11 K. pneumoniae strains carrying the KL64 capsular locus were the most prevalent in the hospital population. The phylogenomic analyses revealed that the metagenome-reconstructed strains and public isolate genomes belonging to the same STs were closely related in the phylogenetic tree. Furthermore, the pangenome structure of the detected K. pneumoniae strains was analyzed, particularly focusing on the distribution of antimicrobial resistance genes and virulence genes across the strains. The genes encoding carbapenemases and extended-spectrum beta-lactamases were frequently detected in the strains of ST11 and ST15. The highest numbers of virulence genes were identified in the well-known hypervirulent strains affiliated to ST23 bearing the K1 capsule. In comparison to traditional cultivation and identification, strain-level metagenomics is advantageous to understand the mechanisms underlying resistance and virulence of K. pneumoniae directly from clinical specimens. Our findings should provide novel clues for future research into culture-independent metagenomic surveillance for bacterial pathogens.

**IMPORTANCE** Routine culture and PCR-based molecular testing in the clinical microbiology laboratory are unable to recognize pathogens at the strain level and to detect strain-specific genetic determinants involved in virulence and resistance. To address this issue, we explored the strain-level profiling of K. pneumoniae prevalent in China based on metagenome-sequenced patient materials. Genome biology of the targeted bacterium can be well characterized through decoding sequence signatures and functional gene profiles at the single-strain resolution. The in-depth metagenomic analysis on strain profiling presented here shall provide a promising perspective for culture-free pathogen surveillance and molecular epidemiology of nosocomial infections.

## INTRODUCTION

Klebsiella pneumoniae is a Gram-negative opportunistic pathogen belonging to the family of *Enterobacteriaceae* (1). It is one of the most common etiologic agents of nosocomial infections for hospitalized patients all over the world (2, 3). Among the multidrug-resistant (MDR) *Enterobacteriaceae* isolated in Sri Lanka, K. pneumoniae dominated (80.7%), followed by Citrobacter freundii (7.0%), Escherichia coli (5.3%), Providencia rettgeri (3.5%), Enterobacter cloacae (1.7%), and Klebsiella aerogenes (1.7%) (4). K. pneumoniae that can produce carbapenemases and extended-spectrum beta-lactamases (ESBLs) has been recently judged as a critical threat for public health by the World Health Organization (5, 6). The epidemiology of carbapenemase-producing and ESBL-producing K. pneumoniae has been extensively investigated (7, 8). Previous studies have pointed out that MDR clinical isolates of K. pneumoniae are usually accompanied by high pathogenicity, which can lead to serious systematic infections, including pneumonia, meningitis, urinary tract and bloodstream infections (9, 10). To date, a number of disease-related virulence factors that contribute to K. pneumoniae infections and host immune evasion have been uncovered, e.g., siderophore systems, capsular polysaccharides (CPS), lipopolysaccharides, and fimbriae (10–12).

Whole-genome sequencing (WGS) and high-throughput genomic analyses on hundreds of K. pneumoniae isolates have provided valuable insights into population structure, hypervirulent clones, and resistance mechanisms of this important pathogen (11, 13–15). However, the WGS-based strategy needs conventional culture and not every strains from the targeted bacterial species can be successfully isolated under the clinical setting. It is still difficult for accurate species/strain identification of the K. pneumoniae species complex by bacterial isolation and hospital pathogen assays, like Vitek2 (16). Additionally, PCR-based molecular tests are unable to identify emerging sequence signatures in evolving pathogens (17). Due to the limited abilities of traditional methods for detecting the clinically relevant genotypes of virulence and resistance, metagenomic next-generation sequencing (mNGS) is becoming an auxiliary technique for cultivation-free and unbiased pathogen detection in hospitalized patients with complicated infections (18, 19). The state-of-the-art computational methodologies on assembly-free metagenomics have enabled profiling of putative bacterial strains and their functional potential at single-strain resolution (20–22). For instance, Kleborate developed by Lam et al. has been applied to gut metagenomes for detecting genotype characteristics that are clinically relevant to K. pneumoniae and other members belonging to the species complex (23).

In this study, our aim is to uncover the molecular characterizations of K. pneumoniae strains directly from the metagenome-sequenced specimens. Strain-level population genomics analyses were performed to identify prevalent sequence signatures and phylogenetic relationships of metagenome-recovered K. pneumoniae strains. Furthermore, the pangenome structure and function of these K. pneumoniae strains were investigated, particularly focusing on the distribution of antibiotic resistance genes and virulence genes across the strains.

## RESULTS

### General features of metagenome-reconstructed strains.

We initially investigated 150 clinical specimens that were subject to mNGS and were positive for K. pneumoniae. Based on species-specific multilocus sequence type (MLST) from metagenomic data, 30 K. pneumoniae strains were detected and designated as metagenome-reconstructed strains (MRSs) herein (Table 1). The sequencing depth of the MRSs was ranged from 5- to 107-fold, with a median depth of 22-fold (Table S1). The reconstructed strains of K. pneumoniae were assigned to 14 different sequence types (STs). Using reference-guided read recruitment and local assembly, 14 capsule types of K. pneumoniae were predicted for the 25 MRSs (Table 1). The most prevalent sequence type was ST11 of nine K. pneumoniae strains, six out of which encoded the CPS loci of KL64. The samples consisting of the ST11 MRSs were distributed in Shanghai (5), Zhejiang (3), and Heilongjiang (1), respectively (Table 1). The second prevalent sequence type was ST15 of five K. pneumoniae strains belonging to KL19 (4) and KL8 (1). The samples of the ST15 MRSs were distributed in Shanghai (4) and Heilongjiang (1). Besides, the taxonomic profiling of species relative abundances showed that K. pneumoniae was the highly abundant bacterial species dominating the communities of the 30 specimens, with a mean abundance of 82%.

**TABLE 1:** Summary of sequence signatures and gene families of K. pneumoniae strains in the metagenomic samples and study participants

Strain	Patient ID	Province^*a*^	RA (%)^*b*^	ST	K type^*c*^	Total genes	Accessory genes	Virulence genes	Resistance genes
Kpn01	#023	Heilongjiang	98.77	11	NA	5,510	2,527	324	37
Kpn02	#008	Shanghai	94.21	11	KL64	5,310	2,327	318	32
Kpn03	#004	Shanghai	99.90	11	NA	5,272	2,289	326	28
Kpn04	#108	Shanghai	70.05	11	KL64	5,536	2,553	330	34
Kpn05	#114	Zhejiang	96.44	11	KL64	5,395	2,412	316	36
Kpn06	#032	Zhejiang	99.69	11	KL64	5,525	2,542	329	33
Kpn07	#019	Shanghai	97.85	11	NA	5,629	2,646	331	35
Kpn08	#088	Zhejiang	100.00	11	KL64	5,480	2,497	329	31
Kpn09	#112	Shanghai	99.76	11	KL64	5,420	2,437	326	33
Kpn10	#042	Shanghai	94.06	15	KL19	5,138	2,155	319	32
Kpn11	#125	Shanghai	75.75	15	KL19	5,159	2,176	318	34
Kpn12	#090	Heilongjiang	90.21	15	KL8	5,107	2,124	300	44
Kpn13	#022	Shanghai	30.72	15	KL19	5,149	2,166	317	36
Kpn14	#079	Shanghai	92.81	15	KL19	5,149	2,166	319	37
Kpn15	#021	Heilongjiang	76.68	23	KL1	5,110	2,127	351	29
Kpn16	#089	Heilongjiang	98.78	23	KL1	5,147	2,164	350	27
Kpn17	#017	Zhejiang	81.75	29	KL54	5,158	2,175	337	29
Kpn18	#052	Shanghai	89.36	29	NA	5,240	2,257	325	28
Kpn19	#059	Zhejiang	91.40	45	NA	4,992	2,009	314	36
Kpn20	#020	Heilongjiang	78.06	45	KL24	5,122	2,139	318	40
Kpn21	#055	Shanghai	42.90	147	KL125	5,184	2,201	309	46
Kpn22	#127	Shanghai	38.87	258	KL107	5,475	2,492	312	40
Kpn23	#049	Heilongjiang	98.85	375	KL2	5,048	2,065	317	27
Kpn24	#041	Heilongjiang	95.39	412	KL57	5,050	2,067	308	28
Kpn25	#018	Zhejiang	66.33	412	KL57	4,984	2,001	306	29
Kpn26	#006	Shanghai	81.21	656	KL149	5,150	2,167	316	45
Kpn27	#061	Shanghai	92.68	660	KL16	5,001	2,018	330	27
Kpn28	#074	Heilongjiang	57.50	902	KL125	5,579	2,596	303	48
Kpn29	#050	Shanghai	89.25	1,049	KL5	5,009	2,026	322	27
Kpn30	#095	Shanghai	38.24	2,471	KL53	4,818	1,835	264	37

a The province information of the clinical samples from the three hospitals are shown: Shanghai for Ruijin Hospital, Zhejiang for Ningbo Women and Children’s Hospital, and Heilongjiang for the Second Affiliated Hospital of Harbin Medical University.

b The percentage relative abundance denotes the estimated proportion of K. pneumoniae in the bacterial community.

c The strains missing the known K types predicted by Kaptive are denoted by NA.

### Population-scale phylogeny of K. pneumoniae.

**FIG 1:**
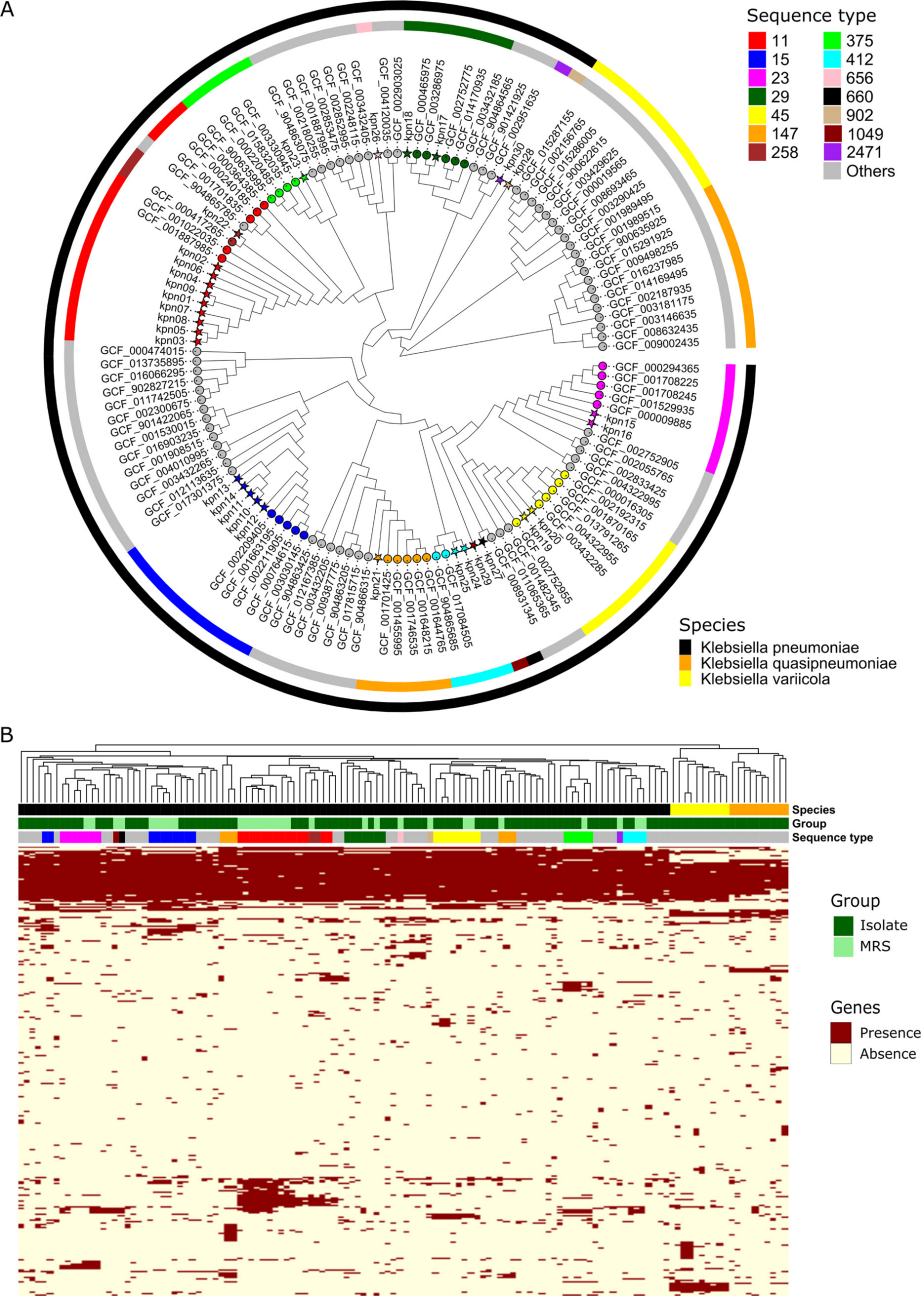
Phylogenomic and pangenomic structure of K. pneumoniae. (A) Maximum likelihood phylogeny of K. pneumoniae. The phylogenetic tree was built using the 38 K. pneumoniae-specific marker genes detected in the 30 metagenomic samples and 100 reference genomes from three major members of the K. pneumoniae species complex. (B) Gene family profiles of K. pneumoniae strains from metagenomes as well as isolate genomes. The heatmap displays the presence/absence patterns of the accessory genes across data sets.

To investigate phylogenetic relationships among bacterial strains, 100 complete isolate genomes of K. pneumoniae species complex were retrieved and compared with 30 metagenome-recovered strains. Using StrainPhlAn, 38 K. pneumoniae-specific marker genes were detected in all the strains. Based on the alignment of single-nucleotide variants (SNVs) in the markers, Fig. 1A displays the phylogenetic tree of the MRSs together with cultivated strains from K. pneumoniae species complex. Obviously, all the metagenome-recovered strains are distributed in the clade of K. pneumoniae and they are distant from the strains in the lineages of *K. quasipneumoniae* and *K. variicola*. It was also observed that the metagenome-recovered K. pneumoniae strains and cultivated strains that shared the same STs were more closely related to each other in the phylogenetic tree. For instance, five ST15 MRSs were placed together with the other cultivated strains of ST15 K. pneumoniae in a single clade without the strains from other STs. Two ST412 MRSs and another two cultivated strains of ST412 K. pneumoniae were placed into a single clade. It suggested that MRSs assigned to the identical STs can be grouped into a single phylogenetic lineage, which also comprised the whole-genome sequenced isolates with the related STs, implying that culture-independent and assembly-free metagenomic analyses would be an alternative to genomic surveillance and epidemiology of K. pneumoniae (23).

### Pangenome structure and function of K. pneumoniae.

To better understand the functional potential of bacterial strains in the community, the pangenome analysis was carried out for decoding gene compositions of individual K. pneumoniae strains in the metagenomic samples. Based on the custom K. pneumoniae pangenome consisting of 24,476 gene families, strain-specific gene repertoires were reconstructed for the MRSs detected above. A total of 9,783 gene families were identified in the pangenome of 30 K. pneumoniae MRSs (Table S3). The number of gene families across the strains was ranged from 4,818 to 5,629 (Table 1). Of these, 2,983 were the core gene families present in all the metagenome-recovered strains and cultivated strains. It was apparent that the dendrogram based on genic components can also cluster all the metagenome-recovered strains into the lineage of K. pneumoniae (Fig. 1B). Besides, all the metagenome-recovered strains could be clustered with the cultivated K. pneumoniae strains affiliated to the same STs, implicating these strains may possess similar phenotypic characteristics.

**FIG 2:**
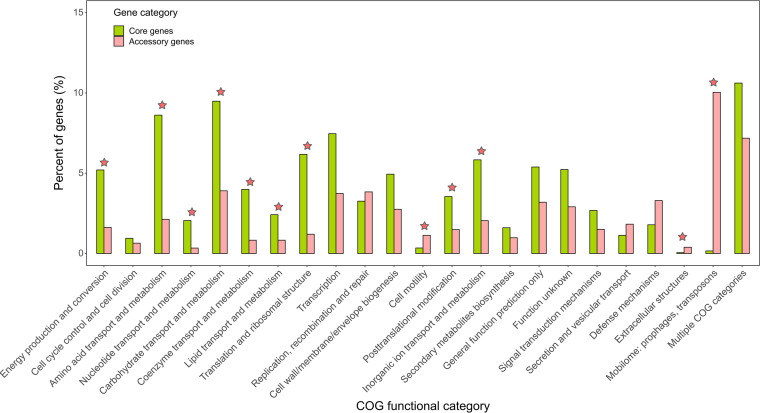
Comparison of COG functional categories between core and accessory gene families in the pangenome of K. pneumoniae MRSs. The asterisk denotes a significant difference in the corresponding category between two genic groups (FDR < 0.001; chi-square test).

Next, the core and accessory gene families of K. pneumoniae strains present in the metagenomes were classified based on COG functional categories (Table S4). As shown in Fig. 2, nine COG categories were significantly abundant in the set of core genes compared with the set of accessory genes (FDR < 0.001). Among these categories, several were associated with basic cellular activities for bacterial growth and survival, for instance, “carbohydrate transport and metabolism” (OR = 2.43), “amino acid transport and metabolism” (OR = 4.05), “energy production and conversion” (OR = 3.21), and “translation and ribosomal structure” (OR = 5.15). On the contrary, three COG categories were significantly abundant in the set of accessory genes (FDR < 0.001), including “mobilome: prophages, transposons” (OR = 0.02), “cell motility” (OR = 0.30), and “extracellular structures” (OR = 0.14). For the genes encoding hypothetical proteins without homologs in the COG database, significant enrichment was found in the set of accessory genes (OR = 0.17) (Table S4).

Besides, functional annotations of the pangenome gene families were performed for antimicrobial resistance (AMR) genes and virulence-associated genes, respectively. The medium number of AMR genes was 33 with a range from 27 to 48 across the metagenome-recovered strains (Table 1). The most abundant AMR genes were found in the strain Kpn28 belonging to ST902. Additionally, the medium number of virulence genes was 318. The highest numbers of virulence genes were observed in both strains Kpn15 and Kpn16 belonging to ST23 and KL1 K. pneumoniae.

### Gene patterns of AMR.

**FIG 3:**
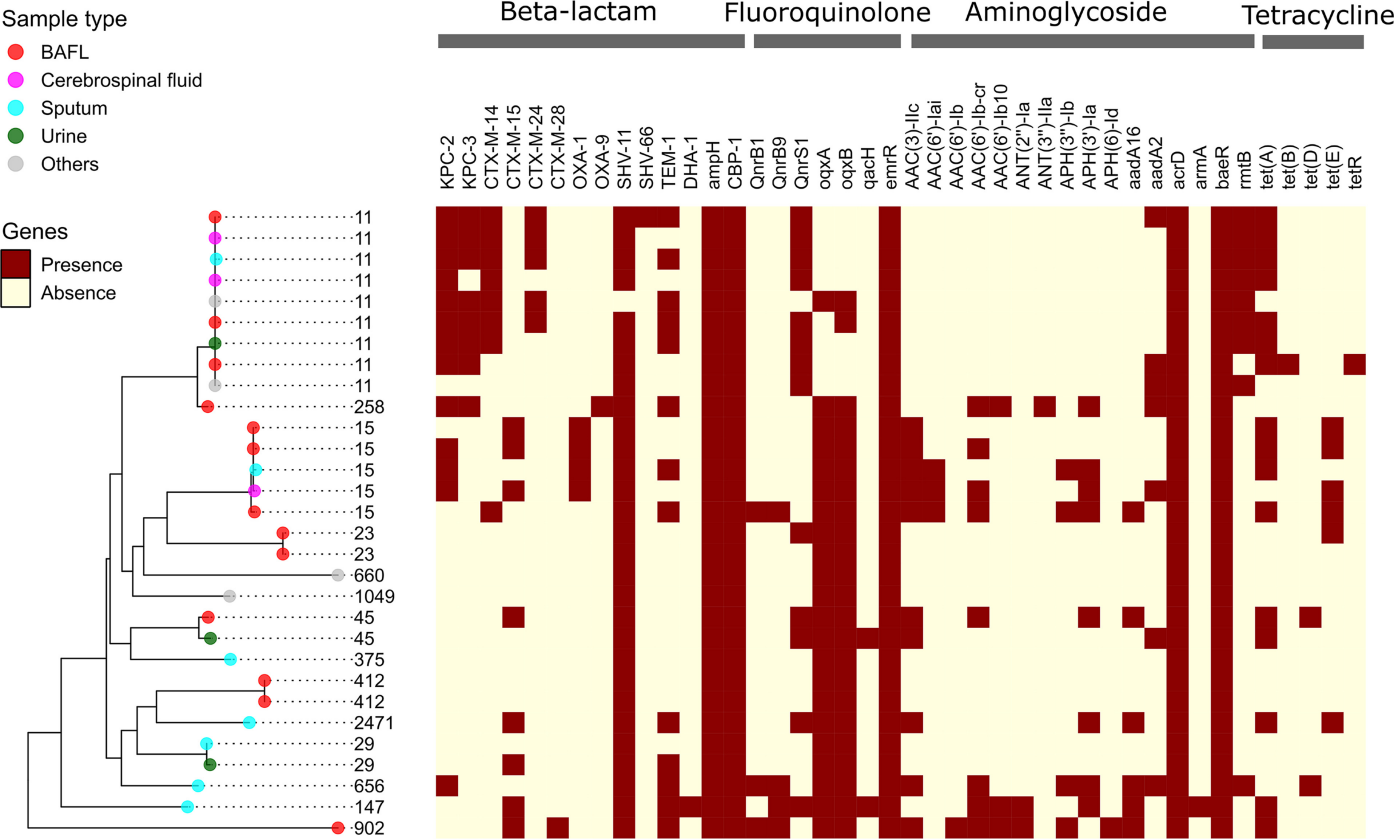
Distribution of antimicrobial resistance (AMR) genes across the metagenome-reconstructed strains of K. pneumoniae. The prediction of AMR genes was performed using RGI searching against CARD. The heatmap shows the presence/absence patterns of the genes conferring resistance to β-lactams, fluoroquinolones, aminoglycosides, and tetracyclines. The list of all detected AMR genes is summarized in Table S5.

Extensive resistance to common antibiotics has been frequently reported in the infections caused by multidrug-resistant strains of K. pneumoniae (24, 25). In this study, we identified the presence and absence patterns of 77 AMR genes across 30 K. pneumoniae MRSs. These AMR genes were assigned to 36 CARD gene families (Table S5). Approximately three quarters (56 out of 77) of all AMR genes were affiliated to the set of accessory genes. Fig. 3 displays the distribution of the AMR genes associated with four classes of antibiotics (i.e., beta-lactams, fluoroquinolones, aminoglycosides, and tetracyclines) across the strains. Among the genes conferring resistance to carbapenems, both genes *bla*_KPC-2_ and *bla*_KPC-3_ encoding carbapenemases were detected in most of the strains affiliated to ST11 (7/9) and in one ST258 strain. *bla*_KPC-2_ was also found in the strains of ST15 (3/5) and ST656 (1/1). Four gene variants of CTX-M beta-lactamases, which are among the most important ESBLs (26), were detected in 16 strains (53.3%). The gene *bla*_SHV-11_ encoding a broad-spectrum beta-lactamase was present in all strains except for a ST11 strain. The mosaic distribution of AMR genes may confer diversified resistance phenotypes to clinical strains of K. pneumoniae.

### Gene patterns of virulence factors.

**FIG 4:**
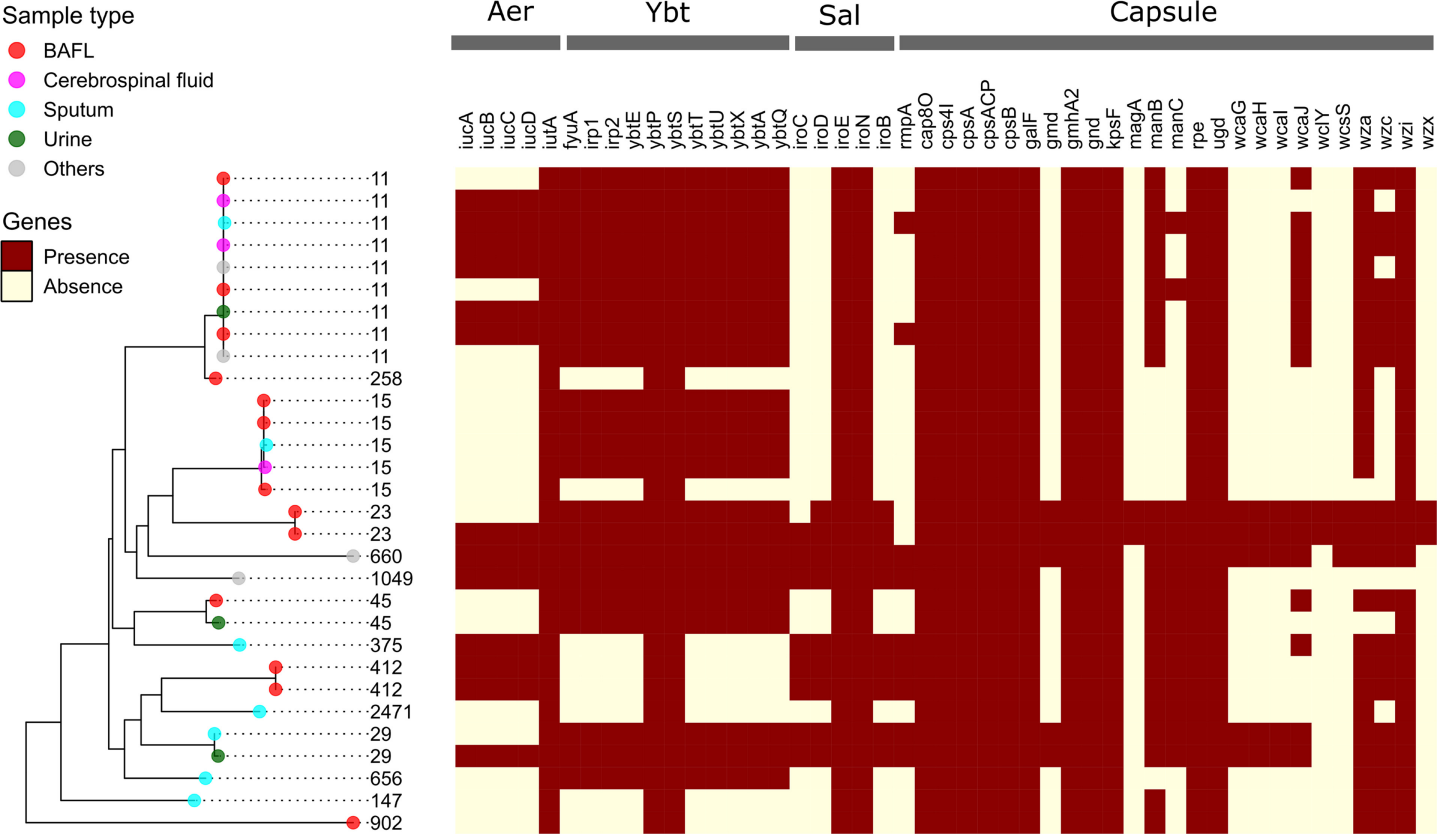
Distribution of virulence genes across the metagenome-reconstructed strains of K. pneumoniae. The prediction of virulence genes was performed using BLAST searching against VARD. The heatmap shows the presence/absence patterns of the genes associated with the biosynthesis of polysaccharide capsule and three siderophores aerobactin (Aer), yersiniabactin (Ybt), and salmochelin (Sal). The list of all detected virulence genes is summarized in Table S6.

In addition to antimicrobial resistance functions, we also investigated the genetic diversity of virulence determinants of K. pneumoniae strains in the metagenomic samples. Herein, 399 virulence-associated genes encoding a variety of bacterial virulence factors were identified and the details are summarized in Table S6. Fig. 4 shows the distribution of the virulence genes coding for products involved in the biosynthesis of iron-scavenging siderophores (i.e., aerobactin, yersiniabactin, and salmochelin) and capsular polysaccharides. The *ybt* locus consisting of 11 genes involved in the synthesis of yersiniabactin, which is the best-known K. pneumoniae high-virulence determinant associated with bacteremia and tissue-invasive infections (11, 27), was found in 22 metagenome-recovered strains of ST11 (9/9), ST15 (4/5), ST23 (2/2), ST29 (2/2), ST45 (2/2), ST660 (1/1), ST656 (1/1), and ST1049 (1/1). The genes *iucABCD* and *iutA* encoding aerobactin were identified in 13 strains of ST11 (6/9), ST412 (2/2), ST23 (1/2), ST29 (1/2), ST375 (1/1), ST660 (1/1), and ST1049 (1/1). The genes *iroBCDE* and *iroN* encoding salmochelin were identified in the strains of ST412 (2/2), ST23 (1/2), ST29 (2/2), ST375 (1/1), ST660 (1/1), and ST1049 (1/1). The genes responsible for the production of enterobactin were identified in nearly all the K. pneumoniae MRSs. Notably, the colibactin synthesis locus *clb* (including 18 genes), which is adjacent to the *ybt* locus in the integrative conjugative elements of K. pneumoniae (28), was only present in the two ST23 strains but absent in the metagenome-recovered strains of other STs. Moreover, both ST23 strains encoded an intact KL1 gene cluster for the production of hypercapsule associated with hypervirulent K. pneumoniae (hvKP) strains (10), including *cpsA*, *galF*, *gmd*, *gnd*, *magA*, *manBC*, *ugd*, *wcaGHIJ*, *wza*, *wzc*, *wzi*, and *wzx* (Fig. 4). Besides, the plasmid-borne gene *rmpA* coding for the regulator of mucoid phenotype A involved in the increase of capsule production was detected in nine metagenome-recovered K. pneumoniae strains of ST11 (2/9), ST412 (2/2), ST29 (2/2), ST375 (1/1), ST660 (1/1), and ST1049 (1/1). Eight out of nine strains carrying the *rmpA* gene also possessed the *Aer* locus (Fig. 4), both of which have been characterized as indicators for the presence of K. pneumoniae virulence plasmids in hypervirulent strains (9, 14). The presence/absence patterns of the above virulence-associated genes in certain lineages should keep in line with the previous studies on the K. pneumoniae clinical isolates and more details are discussed below.

## DISCUSSION

As is well known, bacterial infections caused by K. pneumoniae pose a great threat to global public health. Particularly, high pathogenicity and MDR have brought severe challenges to clinically microbiological testing and anti-infection therapy. High-throughput genomic analyses are beneficial for understanding the genetic diversity of clinically relevant genotypes of virulence and drug resistance during spatiotemporal transmission and adaptive evolution of K. pneumoniae. To address these issues, hundreds of studies on bacterial genomics have adopted culture-dependent whole-genome sequencing, which can produce longer reads and deeper genome coverage for high-quality *de novo* assembly and gene annotation. To explore the application of mNGS to population genomics, we performed integrative analyses to associate K. pneumoniae sequence types or K types with bacterial phylogeny, AMR genes, and virulence genes based on metagenomic sequencing of clinical specimens. Innovative strategies by combining assembly-free and reference-guided local assembly approaches were applied to study strain-level genomics of K. pneumoniae in the clinical setting for the first time.

Thirty K. pneumoniae strains belonging to 14 sequence types were reconstructed based on metagenomic sequencing data in this study. The strain-profiling approaches StrainPhlAn and PanPhlAn were both able to detect and characterize the strains with low sequencing depth even at 5-fold. Our findings further indicated that the distribution of bacterial sequence signatures (i.e., STs and K types) recovered from metagenomes should be well comparable with prior knowledge on the whole-genome-sequenced clinical isolates of K. pneumoniae (15). On the other hand, *rpoB* has been reported to be a species-specific marker for identification of K. pneumoniae isolates (29). Here, the sequences of the *rpoB* gene fragments were available for 14 out of 30 specimens and the blast analysis indicated that the amplified *rpoB* genes belonged to K. pneumoniae (data not shown). Although PCR-based molecular tests and conventional phenotypic methods can support the identification of the targeted species, both exhibit limited utility for providing additional information on clinically relevant lineages (ST) and genotypes. Our study confirms some recent options that metagenomic approaches have enabled culture-free and assembly-free strain profiling analyses for surveillance of the high-risk hvKP clones (20, 22, 30), like ST11, ST15, ST23, ST29, and ST412 detected in the above analysis.

During the past decade, a high prevalence of ST11 carbapenem-resistant K. pneumoniae (CRKP) strains has been reported in the community-acquired and nosocomial infections in China (31–33). Consistently, the most abundant sequence type among all the K. pneumoniae MRSs was found to be ST11, most strains of which encoded the genes *bla*_KPC-2_, *bla*_SHV-11_, *bla*_CTX-M_, *qnrS1*, and *tet(A)* mediating resistance to beta-lactams, fluoroquinolones, and tetracycline (Fig. 3). The co-occurrence of these AMR genes has been recognized in an outbreak of 40 CRKP isolates (34). In addition, the analyses of the local assembly and typing of capsular locus demonstrated that all ST11 K. pneumoniae MRSs possessed a single K type KL64, which has been characterized as a newly emerging superbug prevalent in China by a large-scale genome sequence analysis of 364 ST11 isolates (15). Zhou et al. have also pointed out that, among the ST11 population, KL64 replaced KL47 and became the dominant CRKP clone in China since 2016 (35). Furthermore, the other K type KL105 frequently associated with ST11 was absent in the three representative hospitals in China but was prevalent in Poland and Slovakia (23). The evidence again supports the metagenomic approach for surveillance and epidemiology of K. pneumoniae infections.

Besides, the MDR hypervirulent ST23 K. pneumoniae, whose rapid dissemination is driven by diverse plasmids harboring virulence and ARM genes, is another clinically significant lineage that has been paid close attention to by the medical community (13, 36–38). Meanwhile, the highest numbers of virulence genes were observed in the two ST23 strains (Kpn15 and Kpn16) carrying KL1, a well-characterized capsule type highly associated with hvKP strains (39). It was noted that the most abundant genes involved in synthesizing diverse siderophores (i.e., yersiniabactin, aerobactin, salmochelin, colibactin, and enterobactin) were identified in the Kpn16 strain, perhaps playing roles in bacterial hypervirulence and postinfection proliferation for overcoming iron limitations *in vivo*. Enrichment of these siderophore-related genes in the ST23 lineage has been revealed by a recent study on comparative genomics of K. pneumoniae (11). In particular, the colibactin synthesis locus *clb* present in the metagenome-recovered strains belonging to ST23 has been detected in 3.5% to 4% of K. pneumoniae, in which colibactin production enables genotoxic effect on host cells by inducing double-strand DNA breaks (28, 36, 40).

In summary, we carried out comprehensive strain-profiling analyses to uncover bacterial sequence types, phylogeny, and pangenomic structure of K. pneumoniae recovered from clinical mNGS data. Genome biology of 30 K. pneumoniae strains was characterized by multilocus sequence typing, phylogenetic reconstruction, and capsule typing. Furthermore, the pangenome structure of metagenome-recovered K. pneumoniae strains was analyzed, particularly the distribution of antimicrobial resistance genes and virulence genes across the strains. Our findings should also provide novel clues for future applications of mNGS to molecular epidemiology and culture-free genomic surveillance of clinically relevant pathogens.

## MATERIALS AND METHODS

### Clinical specimens.

In this study, we retrospectively investigated 150 clinical samples that were positive for K. pneumoniae according to mNGS testing. The samples were collected from patients in Ruijin Hospital, Ningbo Women and Children’s Hospital, and the Second Affiliated Hospital of Harbin Medical University from August 2020 to May 2021. The information of all samples was listed in Table S1. Ethical approval for the study was obtained from the local Medical Ethics Committee of the Ruijin Hospital (Approval ID KY2021-213).

### Metagenomic sequencing and data preprocessing.

The experiments of mNGS were carried out at Genoxor Inc., China. Microbial DNA was extracted and enriched by streamlined host DNA depletion using HostZERO Microbial DNA Kit (Zymo, United States). Extracted DNA was sheared to 300 bp fragments with Covaris M220 (Covaris, MA, United States) following the manufacturer’s protocol. Metagenomic libraries were then constructed using the NEBNext Ultra DNA Library Prep Kit for Illumina. Multiplexed libraries were sequenced in a 75-bp single-end mode using a NextSeq 550 system (Illumina Inc., USA). Raw sequencing data were demultiplexed into Fastq-formatted reads using bcl2fastq v2.20 (Illumina). Trimming adaptor sequences and filtering low-quality bases/reads were then performed using Trimmomatic v0.36 with the options LEADING:15 TRAILING:15 SLIDINGWINDOW:5:20 MINLEN:36 AVGQUAL:20 (41). Human-derived DNA contamination was subtracted through aligning reads to the human reference genome GRCh37 using Bowtie v2.2.6 with the options –threads 32 –end-to-end (42). Species identification of K. pneumoniae was performed by Kraken v2.0.9 (43). Sequencing depth and genome coverage were estimated by mapping reads to the complete genome of K. pneumoniae HS11286 using BBmap v38.18 (44). To estimate the relative abundance of K. pneumoniae reads in the bacterial community, the taxonomic profile of species abundance was calculated using Bracken v2.2 (45).

### Analyses of MLST and capsule serotype.

For strain identification and typing of K. pneumoniae from the metagenomic sample, the MLST was detected using the pipeline metaMLST v1.2.2 (46). Briefly, the metaMLST database curated from pubMLST (47) was used to generate a bowtie2 database of K. pneumoniae allelic sequences from seven housekeeping genes: *gapA*, *infB*, *mdh*, *pgi*, *phoE*, *rpoB*, and *tonB*. The consensus sequences of K. pneumoniae MLST loci present in the metagenomic sample were reconstructed by read alignment against the bowtie2 database and then by a majority rule consensus approach implemented by the mpileup utility in the SAMtools package v0.1.19 (48). The resulting allele sequences were used to assign known and novel ST numbers to individual samples according to the organism-specific MLST protocol. The strains assigned with known STs were defined as the MRSs in the individual samples.

The capsule serotype (K type) was determined by an integrative pipeline based on read recruitment, local assembly, and capsule typing. Briefly, metagenomic reads per sample were first recruited to the Klebsiella capsule locus reference database in the Kaptive package v0.7.3 (49) using BBmap v38.18. Only mapped reads were extracted to assemble the capsule locus using Megahit v1.2.9 (50). The K type was predicted for the resulting genomic assembles using Kaptive (49).

### Strain-level profiling analyses.

We employed StrainPhlAn (21), a strain-level profiling tool integrated with the pipeline MetaPhlAn v3.0, for tracking targeted strains in the clinical samples. Briefly, Bowtie2 was used to align metagenomic reads to the MetaPhlAn marker database comprising of ∼1.1 million unique clade-specific marker genes from ∼17,000 species (51). The resulting SAM files were used to reconstruct the sequences of marker genes from all species strains in each sample. Additionally, 46 K. pneumoniae-specific marker genes were extracted from the MetaPhlAn database for detection of the corresponding genes in the reference genomes of isolates using blast v2.10.0 (52). The markers present in the MRSs and isolate genomes were selected and their sequences were concatenated. A multiple sequence alignment was then produced for reconstruction of the maximum-likelihood phylogenetic tree using the package PhyloPhlAn v3.0.60 (53).

On the other hand, PanPhlAn v1.3 (20), which is also a strain-level metagenomic profiling tool, was used to investigate the genic compositions of the MRSs. Firstly, a custom pangenome was created with 100 complete reference genomes derived from three members within the K. pneumoniae species complex, including 80 genomes from K. pneumoniae (KpI), 10 genomes from *K. variicola* (KpII), and 10 genomes from *K. quasipneumoniae* (KpIII). The genome sequences of cultured isolates were downloaded from the NCBI Assembly database. The STs of individual isolates were predicted by using MLST v2.16.2 with the seven housekeeping alleles: *gapA*, *infB*, *mdh*, *pgi*, *phoE*, *rpoB*, and *tonB* (Table S2). The isolate genomes were preferentially selected based on the STs related to the MRSs. Roary v3.13.0 was used for the generation of the PanPhlAn pangenome database of K. pneumoniae. Next, the gene presence/absence patterns of individual strains were scanned by PanPhlAn with the options –min_coverage 2 –left_max 1.25 –right_min 0.75.

### Functional annotation of gene family.

Protein functional classification for the pangenome gene families was conducted based on sequence similarity searching against the Clusters of Orthologous Groups (COG) database (54) with blastp v2.10.0. Annotation of AMR genes was performed using the Comprehensive Antibiotic Resistance Database (CARD) and the related program RGI v5.1.1 (55). The query sequences were annotated by the two RGI paradigms perfect and strict. Annotation of virulence-associated genes was performed using the Virulence Factor Database (VFDB) (56) and blastp v2.10.0. The query sequences were annotated by the top hit with the maximum *E*-value of 1e-20.

### Statistical analyses and data visualization.

Comparison of the gene count data was estimated using odds ratio (OR) and chi-square test implemented by the R package Epitools v 0.5–10.1 (57). The phylogenetic tree integrated with the other metadata (sequence types and taxonomic origins of strains) was visualized using the R package ggtree v3.0.2 (58). Hierarchical clustering on a binary matrix of pangenome gene families across the strains was performed and visualized using the R package ComplexHeatmap v2.8.0 (59). The statistical analyses and data visualization were carried out in R v4.1.0 (60).

### Data availability.

The microbial reads produced in this study have been deposited in the NCBI Sequence Read Archive (SRA) database under the BioProject accession PRJNA758247.
