# Supplementary material for: Metagenomic Approaches Reveal Strain Profiling and Genotyping of Klebsiella pneumoniae from Hospitalized Patients in China
Source: Microbiol Spectr. 2022 Mar 23;10(2):e02190-21. doi: 10.1128/spectrum.02190-21 (PMC9045201; doi:10.1128/spectrum.02190-21)
Supplement: SUPPLEMENTAL FILE 7 — Supplemental material. Download SPECTRUM02190-21_Supp_7_seq14.pdf, PDF file, 0.2 MB [file spectrum02190-21_supp_7_seq14.pdf]

Supplemental Materials

Table S1. Statistics of metagenome sequencing and quality control for the samples used in this study.

Table S2. List of reference genomes of *K. pneumoniae* species complex used in this study.

Table S3. Summary of gene family presence and absence in the *K. pneumoniae* pangenome of 30 metagenome-reconstructed strains.

Table S4. Summary of COG-based functional classification of *K. pneumoniae* pangenome gene families.

Table S5. Distribution of antimicrobial resistance genes in the metagenome-reconstructed strains of *K. pneumoniae*.

Table S6. Distribution of virulence-associated genes in the metagenome-reconstructed strains of *K. pneumoniae*.
